# Economic burden of age-related macular degeneration in routine clinical practice: the RAMDEBURS study

**DOI:** 10.1007/s10792-021-01906-x

**Published:** 2021-06-10

**Authors:** José M. Ruiz-Moreno, Luís Arias, Maximino J. Abraldes, Javier Montero, Patricia Udaondo, The RAMDEBURS study group

**Affiliations:** 1 Puerta de Hierro-Majadahonda University Hospital, Joaquín Rodrigo, 2 Majadahonda, 28222 Madrid, Spain; 2 Department of Ophthalmology, Castilla La Mancha University, Albacete, Spain; 3 Red Temática de Investigación Cooperativa en Salud: “Prevención, detección precoz, y Tratamiento de La Patología Ocular Prevalente, Degenerativa Y Crónica” (RD16/0008/0021), Spanish Ministry of Health, Instituto de Salud Carlos III, Madrid, Spain; 4 Miranza, Spain; 5 Bellvitge University Hospital, Barcelona, Spain; 6 Santiago de Compostela University Hospital Complex, A Coruña, Spain; 7 Río Ortega University Hospital, Valladolid, Spain; 8 University and Polytechnic Hospital of La Fé, Valencia, Spain

**Keywords:** Age-related macular degeneration, Health economics, Economic burden, Vascular endothelial growth factor inhibitors, VEGF

## Abstract

**Purpose:**

To describe and evaluate the main direct health costs, in routine clinical practice, of age-related macular degeneration (AMD) patients, from hospital perspective, in Spain.

**Methods:**

Retrospective, multicenter, and observational study conducted on five third-level Spanish hospitals, between December 2018 and December 2019. The study included patients who were diagnosed of AMD before December 2018. Direct healthcare costs were obtained from a Spanish database. Study variables included demographic and clinical variables, and resources, such as treatment, diagnostic tests, medical examination, and surgery. Among the 1414 screened AMD patients, 1164 patients were included. In the overall study patients, the total cost was €5,386,511.0, with a mean cost per patient of €4627.6 ± 2383.9. The largest cost items were diagnostic examinations (€2.832.902,0) and vascular endothelial growth factor inhibitors (anti-VEGF) treatment (€2.038.257,2). Bevacizumab was administered to 325 (27.9%) patients, ranibizumab to 328 (28.2%), and aflibercept to 626 (53.8%); 115 (10.7%) patients received two anti-VEGF treatments, while 90 (7.7%) did not receive any. Over the course of the study, a total of 6,057 anti-VEGF injections were administered, with a mean (95% confidence interval) of 4.8 (4.4–5.2) injections per patient. Regarding safety, 29 patients experience injection-related adverse events, among them 12 patients had cataract and 11 ones elevated intraocular pressure (IOP). The incidence of endophthalmitis was 0.5% (6/1164).

**Conclusions:**

AMD was associated with considerable healthcare costs for regional healthcare systems. Diagnostic examinations, particularly OCT examinations, and anti-VEGF treatment represented the largest cost items.

**Supplementary Information:**

The online version contains supplementary material available at 10.1007/s10792-021-01906-x.

## Introduction

Age-related macular degeneration (AMD) is a prevalent, chronic, and progressive retinal degenerative disease of the macula [1, 2].

AMD constitutes one of the leading causes of severe and irreversible visual impairment globally, but most notably in developed countries, among the elderly [3–9]. Its overall prevalence is approximately 8.7%, although variation among different populations is substantial [3–9]. The results of a metaanalysis that included 129,664 subjects showed that the prevalence of AMD ranged from 7.3% in Asian population to 12.3% in European ancestry population [5].

Additionally, as the life expectancy is rising up, the importance of AMD increases [10]. It was estimated that the number of people with the disease would be around 196 million in 2020, increasing to 288 million in 2040 [5].

Generally speaking, AMD can be classified as early, intermediate, or late stage [11]. Compared with early AMD, late AMD is far less frequent but most damaging to the sight [11]. According to the latest global estimate of AMD, the prevalence of late AMD in populations of European ancestry was 0.5% (95% confidence interval, CI: 0.26–1.08%) [5].

The information about the prevalence of AMD in Spain is very limited. Based on the currently available evidence, the estimated prevalence of late AMD (either geographic atrophy or macular neovascularization) ranges between 1.1% (95% CI: 1.0–1.2%) [7] and 1.9% [12].

Despite the fact that the introduction of vascular endothelial growth factor inhibitors (anti-VEGF) has supposed a significant advance in therapeutic management of neovascular AMD (NVAMD), none of them cures the disease or reverses its course [13–16]. Additionally, the main drawback of anti-VEGF is their high cost, which suppose a significant burden for health systems, often making such a regimen unaffordable in clinical practice.

According to the results of a metaanalysis, intravitreal aflibercept was associated with a higher overall treatment cost than ranibizumab (18,187 € vs. 17,168€, respectively) [17].

Although bevacizumab has been identified as the most cost-effective treatment, its use is "off-label" and not considered the standard of care for NVAMD in Europe (nor it is approved for treating NVAMD by US or European regulatory agencies) [18].

The Spanish Health Systems are public, universal, and mostly free of charge for the patients except for the share of out-of-pocket expenditure, such as transportation-associated costs, meals, glasses and contact lenses prescription, or medicine co-payment, among others [19].

Because AMD treatment entails a significant impact on the Health System budget, it is extremely important to accurately know the cost of these therapeutic strategies.

This study aimed to describe and evaluate the main direct health costs (monetary value), in routine clinical practice, of AMD patients, from hospital perspective, in Spain. Additionally, this study also assessed different clinical and demographic characteristics of the study population.

## Methods

Retrospective, multicenter, and observational study conducted on patients diagnosed of AMD, who were treated in 5 third-level Spanish hospitals, between December 2018 and December 2019.

The study was conducted in accordance with the tenets of the Declaration of Helsinki. The protocol was approved by the ethics committee of Puerta de Hierro-Majadahonda University Hospital, which waived the need for informed consent for study participation.

### Inclusion/exclusion criteria

This study included patients with a clinical diagnosis of AMD, who were treated in the Ophthalmology department, between December 2018 and December 2019, in one of the five third-level university hospitals that participated in the study. Patients must have been diagnosed of AMD before December 2018.

Those patients with a clinical diagnosis of AMD who did not require medical interventions, either treatments or visits, during the study follow-up were excluded.

### Study centers

Five third-level hospitals (Listed in the Annex I), representing five Spanish Autonomous Communities (in alphabetical order: Castilla y León; Cataluña; Galicia; Madrid; and Valencian Community), were selected to participate in the study.

Each center was represented by a principal investigator and two sub-investigators from the ophthalmology department.

The Research Group was constituted under the name of Real-World Evidence study of patients with Age-Related Macular Degeneration to evaluate the Economic Burden in Spain (RAMDEBURS).

### Costs

Direct healthcare costs were obtained from a Spanish database [20].

The cost of sanitary and consumable supplies, as well as that of antiangiogenic treatments, was provided and averaged by the study centers.

Costs are expressed in euros (€) and have been updated for the year 2020 [20]. An overview of the unit costs is shown in Annex II.

The overall direct healthcare costs were calculated, as well as the mean cost per patient.

Total costs were estimated considering the unit cost of the different resources and the number of resources consumed by each patient.

### Study variables

The information, collected from the medical record, was introduced in an electronic case report form (CRF). For each study participant, the following information was registered:

- Demographic variables: Age, sex, and smoking habit.
- Clinical variables: Type of AMD (exudative, dry, or both); affected area; year of diagnosis; topical prophylactic treatment (before and after intravitreal injections); injection site; type of anesthesia; and injection-related adverse events (cataract, retinal detachment, endophthalmitis, elevated intraocular pressure).
- Resources:
  - Treatment: Anti-VEGF administered (type and number of injections), and sanitary and consumable supplies.
  - Diagnostic examinations: visual acuity (VA), tonometry, optical coherence tomography (OCT), fluorescein angiography (FA), indocyanine green (ICG), autofluorescence, retinography, fundus (indirect ophthalmoscopy), fundus (biomicroscopy), ultrasonography, and genetic tests.
  - Medical examinations: ophthalmology and emergency.
  - Surgery: vitrectomy.

### Statistical analysis

A standard statistical analysis was performed using the MedCalc® Statistical Software version 19.5.3 (MedCalc Software Ltd, Ostend, Belgium; https://www.medcalc.org; 2020).

Descriptive statistics number (percentage), mean [standard deviation (SD)], mean [95% confidence interval (95% CI)], or median (95% CI) were used, as appropriate.

## Results

**Fig. 1:**
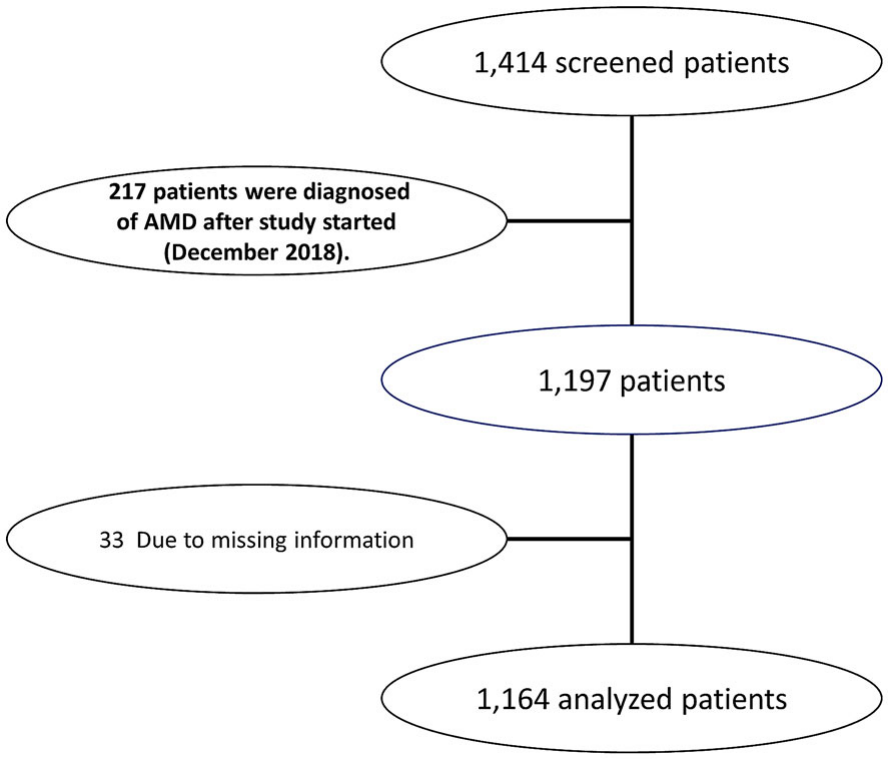
Study flowchart

Among the 1414 screened AMD patients, 1164 patients fulfilled the respective demands of the inclusion and exclusion criteria. Figure 1 shows the study flowchart.

Mean (95% CI) age of study sample was 79.8 (79.3–80.2) years, and 689 (59.2%) were women. Table 1 shows the main demographic and clinical characteristics of the study population.

**Table 1:** Demographic and clinical characteristics

Variable	*n* = 1164
*Age, years*	
Mean (SD)	79.8 (8.2)
Range	44.0–100.0
*Sex, n (%)*	
Woman	689 (59.2)
Man	475 (40.8)
*Smoking habits, n (%)*	
Yes	404 (34.7)
No	94 (8.1)
Former	89 (7.6)
Missing data	577 (49.6)
*Type AMD, n (%)*	
Exudative	1012 (87.0)
Atrophic	12 (1.0)
Both	140 (12.0)
*Affected eye, n (%)*	
One eye	514 (44.2)
Both eyes	650 (55.8)
*Duration, years*	
Mean (SD)	2.7 (2.8)
Range	0.0–24.0
*Prophylactic treatment, n*^*a*^*(%)*	
Before	133 (18.2)
After	574 (81.2)
*Anti-VEGF*^*b,c*^*(%)*	
Bevacizumab	325 (27.9)
Ranibizumab	328 (28.2)
Aflibercept	626 (53.8)
*Place of injection, n*^*b*^*(%)*	
Operating room	134 (11.5)
Clean room	1027 (88.2)
Office setting	3 (0.3)
*Anesthesia, n*^*b*^*(%)*	
Topical	1070 (99.6)
Subconjunctival	4 (0.4)

^a^*n* = 707 subjects

^b^*n* = 1074 subjects

^c^One-hundred and fifteen patients received two anti-VEGF treatments and 90 ones received none

*N* Number; *SD* standard deviation; *CI* confidence interval; *AMD* age-related macular degeneration; *Anti-VEGF* vascular endothelial growth factor inhibitors

Regarding AMD treatment, bevacizumab was administered to 325 (27.9%) patients, ranibizumab to 331 (28.4%), and aflibercept to 626 (53.8%); 115 patients received two anti-VEGF treatments, while 90 did not receive any. Similar proportion of patients received treatment with bevacizumab or ranibizumab (*p* = 0.8302). However, a significant proportion of patients received treatment with aflibercept than with bevacizumab (mean difference 25.9%, 95% confidence interval: 21.9–29.6%, *p* < 0.0001) or ranibizumab (mean difference 25.4%, 95% confidence interval: 21.5–29.2%, *p* < 0.0001).

**Fig. 2:**
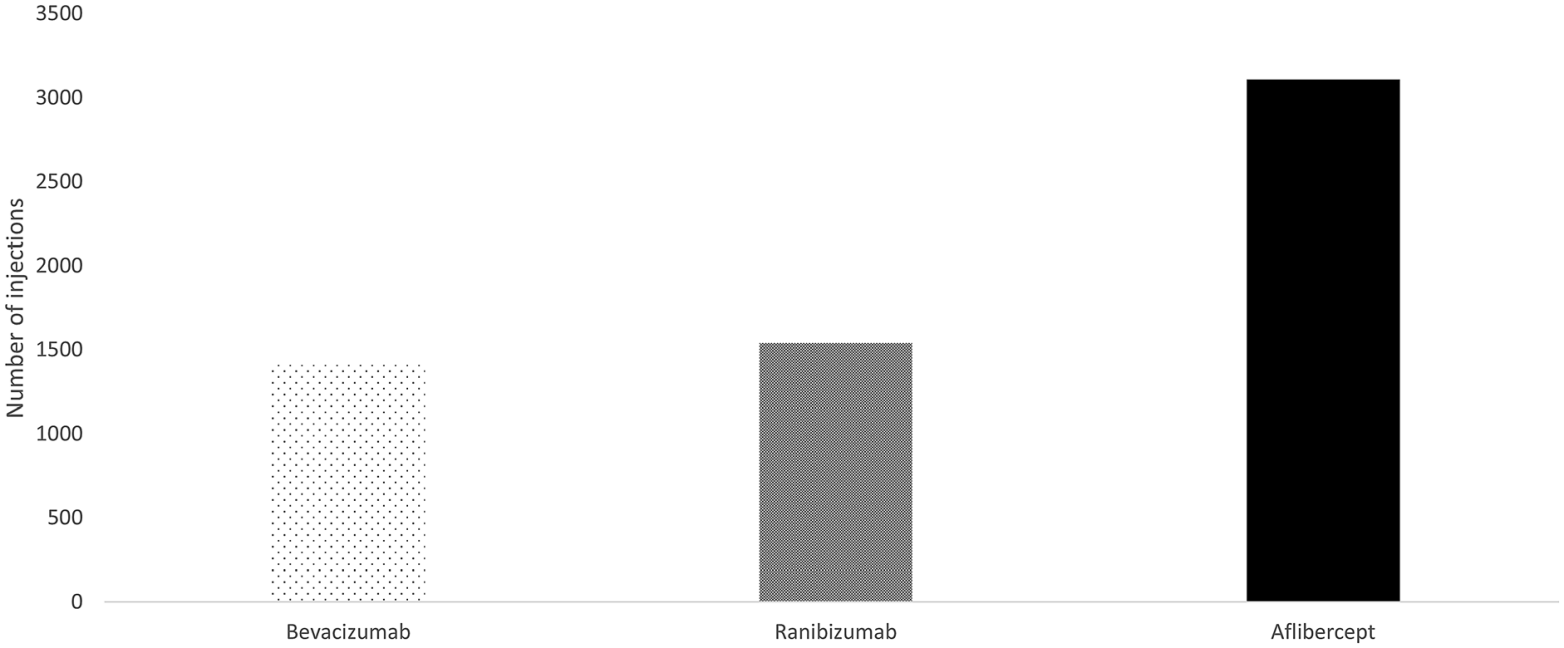
Number of intravitreal injections according to the vascular endothelial growth factor inhibitor administered

Over the course of the study, a total of 6057 anti-VEGF injections were administered, with a mean (95% CI) of 4.8 (4.4–5.2) injections per patient. Figure 2 shows the distribution of the anti-VEGF treatments administered during the study.

In the overall study patients, the total cost was €5,386,511.0, with a mean cost per patient of €4627.6. The largest cost items were diagnostic examinations (€2.832.902,0) and anti-VEGF treatment (€2.038.257,2) (Table 2).

**Table 2:** Overview of the total costs^a,b^

Item	Total, €	Mean (SD)/per patient, €	Range, €
Diagnostic examinations	2,832,902.0	2433.8 (1615.8)	176.1–11.254.8
Medical examinations	503,706.5	432.7 (222.5)	0.0–1659.1
Surgery	11,645.4	10.0 (152.4)	0.0–2329.1
Total	5,386,511.0	4627.6 (2383.9)	253.6–15,600.1

^a^The mean costs were calculated for the total study population (*n* = 1164 patients)

^b^Costs have been updated for the year 2020

*SD* standard deviation; *Anti-VEGF* vascular endothelial growth factor inhibitors

**Fig. 3:**
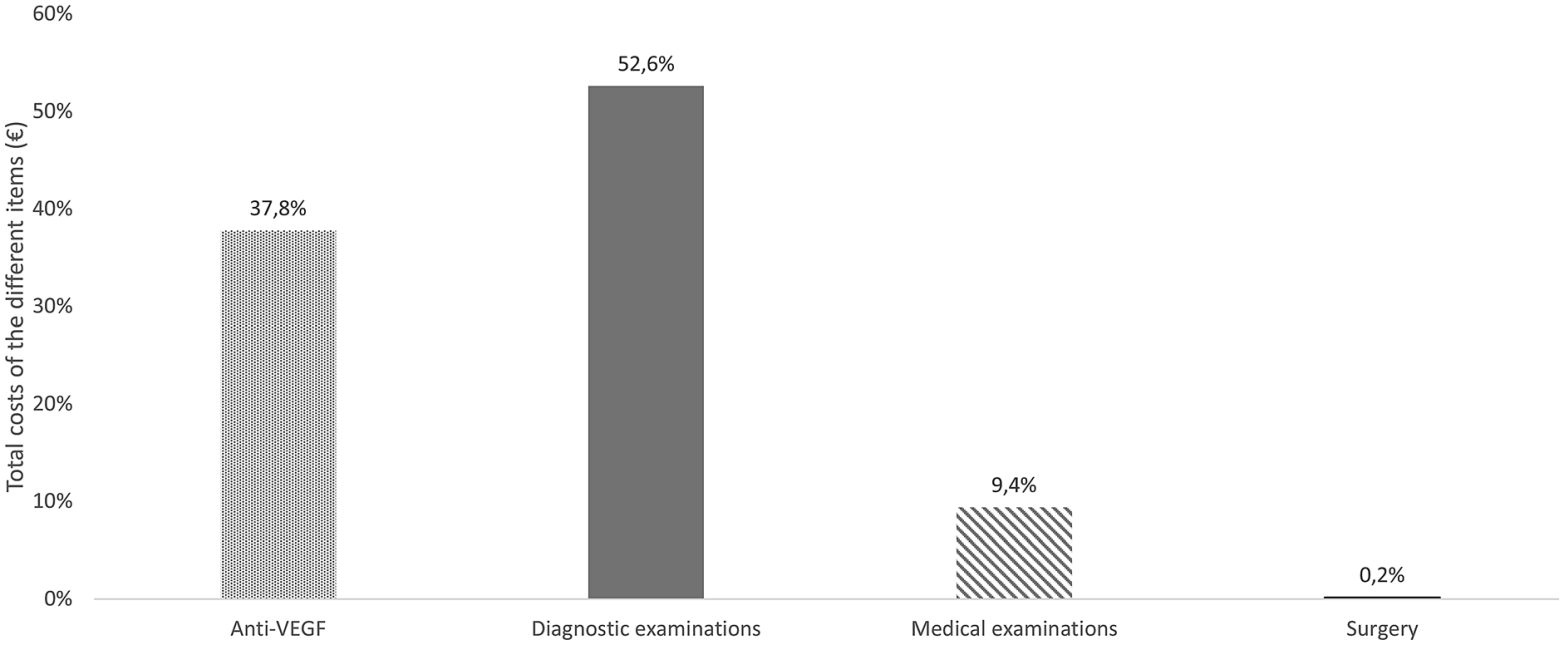
Overview of costs distribution among the different items (%)

The costs of anti-VEGF treatment supposed 37.8% of total direct health costs (Fig. 3).

Among the 1,074 patients who received anti-VEGF treatment, the mean (SD) cost/per patient was €257.8 (162.2), €3525.8 (2242.4), and €1274.8 (718.1) for patients treated with bevacizumab, ranibizumab, and aflibercept, respectively.

In these group of patients, the mean cost per patient was 4727.4 ± 2281.8 €, which was significantly greater than that observed in the 90 patients who did not receive anti-VEGF therapy (2042.6 ± 1307.8 €); mean difference: 2684.8 €; 95% CI: 2206.3–3163.3 €, *p* < 0.0001.

Table 3 summarizes the direct costs of the different items. Among diagnostic examinations, OCT (€1,125,541.5) and retinography (€775,951.7) represented the largest cost items. About examinations costs, ophthalmology examination represented 96.6% of the total amount.

**Table 3:** Overview of breakdown of costs by item^1^

Item	n		Total, €	Mean (SD)/per patient, €	Range, €
Anti-VEGF	1074^2^	Overall	2,038,257.2	1897.8 (1866.1)	0.0–11,271.5
	325	Bevacizumab	83,796.3	257.8 (162.2)	59.4–950.9
	328	Ranibizumab	1,156,450,8	3525.8 (2242.4)	751.4–11,271.5
	626	Aflibercept	798,010.1	1274.8 (718.1)	256.8–4108.2
Diagnostic examinations	1164	Overall	2,832,902.0	2433.8 (1,615.8)	176.1–11,254.8
	1160	OCT	1,125,541.5	970.3 (586.1)	123.o–3198.3
	66	FA	5754.7	87.2 (106.0)	65.4–915.6
	28	ICG	2594.0	92.6 (22.7)	86.5–172.9
	138	Angio-OCT	41,049.0	297.5 (307.5)	136.8–2736.6
	153	Autofluorescence	11,377.1	74.4 (46.9)	39.8–238.7
	652	Retinography	775,951.7	1190.1 (735.0)	140.2–4486.1
	285	Fundus^a^	36,823.0	129.2 (81.3)	15.9–382.1
	860	Fundus^b^	303,286.9	352.7 (239.7)	53.1–1359.3
	1160	VA	404,347.1	348.6 (213.8)	53.1–1359.3
	640	Tonometry	125,251.1	195.7 (160.4)	53.1–1359.3
	3	Genetic test	182.9	61.0 (0.0)	61.0–61.0
	1	Blood analysis	116.9	116.9 (N.A.)	N.A
	2	Visual field	232.4	116.2 (98.6)	46.5–185.9
	2	Ultrasonography	394.0	197.0 (N.A.)	197.0–197.0
Medical examinations	1145^3^	Overall	503,706.5	439.9 (217.2)	0.0–1659.1
	1143	Ophthalmology	486,522.2	425.7 (204.6)	77.5–1549.9
	99	Emergency^4^	17,194.3	173.6 (95.9)	139.7–698.6
Surgery	5	Overall	11,645.4	2329.1 (N.A.)	2329.1–2329.1
	5	Vitrectomy	11,645.4	2329.1 (N.A.)	2329.1–2329.1

^1^Costs have been updated for the year 2020

^2^115 patients received two anti-VEGF and 90 did not receive any

^3^Patients could have gone to both departments

^4^Only ophthalmology emergencies

^a^Indirect ophthalmoscopy

^b^Biomicroscopy

Throughout the study follow-up, 29 patients experience injection-related adverse events (AEs), among them 12 patients had cataract and 11 ones elevated IOP. The incidence of endophthalmitis was 0.099% (95% confidence interval: 0.036–0.215%) per intravitreal injection.

Three patients had more than one AE. An overview of the different treatment-related AEs is shown in Table 4.

**Table 4:** Incidence of treatment-related adverse events (AEs) in the study sample (1164 subjects; 6057 injections) during the study follow-up

Adverse event	Number (%)
Overall	29 (2.5)
Cataract	12 (1.0)
Elevated IOP	11 (0.9)
Endophthalmitis*	6 (0.099)
Retinal detachment	1 (0.1)
Other	3 (0.3)

^*^Per injection (sample 6057 injections)

## Discussion

This study was designed to assess the economic burden, in terms of direct health costs, of AMD in a patient population where the predominant treatment was anti-VEGF therapy.

Late-stage AMD may be divided into two different forms, namely the nonvascular subtype or dry AMD (geographic atrophy) and the neovascular subtype (NVAMD) or wet AMD, which is less frequent but responsible of approximately the 90% of blindness related to AMD [1, 2].

To our knowledge, information evaluating the economic burden of AMD in Spain, since the advent of anti-VEGF therapy as the standard of care, is very limited.

According to the results of this study, diagnostic tests and intravitreal anti-VEGF injections represented the items with the largest direct health costs.

Preserving population health requires work and money. Achieving it implies that National Health Systems should face unlimited demand with limited resources. That is why, health economics is exerting an influence on decision making at all levels of health care [24].

Demographic aging is leading to a substantial increase in the prevalence of age-related sight-impairing conditions and associated increases in their costs [10, 25, 26]. However, despite the relevance of this issue, to date, there has been little work evaluating the economic impact of AMD in a Spanish setting.

The average annual societal cost per bilateral NVAMD patient treated was estimated to be euro 5732 in Spain, direct vision-related medical costs accounted for 23–63% of the total cost [27]. When we update the prices by using the cost price index (CPI), it results in an increase of the 14.8% between January 2008 and July 2020 [28]. With this rate of variation, the updated costs of Cruess et al. [27] are €6,534.5. In our study, mean annual cost/per patient was slightly lower (€4,627.6), but we did not consider direct non-medical-related costs, like, for example, home healthcare and social services costs.

According to data of National Statistics Institute, there are about 9.27 million people ≥ 65 years in Spain [29]. The prevalence of NVAMD in Europe, among subjects ≥ 65 years, has been estimated to be 2.29% [27]. Based on this assumption, there are approximately 212,280 patients (≥ 65 years) with NVAMD in Spain [29, 30]. Based on this estimation, the main direct health costs associated with NVAMD might suppose €982.4 million. On the other hand, the Spanish Eyes Epidemiological (SEE) Study estimated an overall prevalence of AMD of 3.4% among subjects ≥ 65 years [12]. Assuming these figures, approximately 315,180 patients (≥ 65 years) would have NVAMD in Spain, which suppose €1458.5 million. In summary, it is possible to estimate that the total direct health burden associated with NVAMD (main direct health costs per patient x estimated number of Spanish patients with NVAMD) would range between €982.4 million and €1458.6 million, which represents an 1.37–2.10% of Spanish total public health spending [31].

Although medical treatment of NVAMD experienced a significant advance due to the introduction of anti-VEGF agents, they have several drawbacks, including their high cost and the lack of efficiency.

Among patients included in the current study, 27.9% received treatment with bevacizumab, 28.2% with ranibizumab, and 53.8% with aflibercept. Although, when compared to Italy, the proportion of patients treated with bevacizumab was similar, the proportion of patients treated with ranibizumab and aflibercept was totally different [32]. While in the current study 53.8% of patients were treated with aflibercept, in Italy only 25.3% of patients received treatment with it. Similarly, 28.2% of patients received treatment with ranibizumab, while that proportion was 55.3% in Italy [32].

The cost differences between ranibizumab and aflibercept may be due to the fact that costing approach assumes vial splitting for aflibercept, but not ranibizumab. Although preloaded ranibizumab is usually the first option, in those cases that the ophthalmologist decides to use a vial, vial splitting, as performed with aflibercept, would be used for ranibizumab as well.

Despite anti-VEGF therapy has become the current standard of care for NVAMD [33], many patients do not respond adequately to this therapy or experience a slow loss of efficacy of anti-VEGF agents after repeated administration over time [34, 35].

Although new approaches for treating NVAMD have been proposed, as far as we know, there is no evidence about their cost or cost-effectiveness.

Regarding complications, the high incidence of endophthalmitis reported in the current study is noteworthy. On average, the incidence of endophthalmitis after intravitreal injections of anti-VEGF or corticosteroids is low [36–41]. It ranges between 0.00% [41] and 0.021% [36]. In our study, the incidence of endophthalmitis was 0.099% (95% CI: 0.036–0.215%). Although the incidence of endophthalmitis was slightly greater than that reported in other studies, there is no any objective reason, with the exception of the small sample size, that might justify this finding.

It is important to acknowledge some limitations when interpreting these findings. Our study did not evaluate direct non-medical-related costs (e.g., home healthcare and social services), patient transportation, or other incidentals, to establish economic parameters. The study sample was limited to five Spanish Autonomous Communities, which may only reflect the reality of these regions. Nevertheless, the methodology could be easily replicated in other regions. Finally, it should be mentioned that mean cost per patient in the overall study sample was affected by the fact that 90 patients did not receive anti-VEGF therapy. Nevertheless, the mean cost per patient in eyes who underwent anti-VEGF therapy and those who did not was calculated, which solves this limitation.

## Conclusions

NVAMD was associated with considerable healthcare costs. Diagnostic examinations, particularly OCT examinations, represented the largest cost item.

Additionally, anti-VEGF treatment represented a relevant burden for healthcare systems, due mainly to its high price, needs for repetitive administration, and frequent outpatient visits.

Further studies are needed to determine the role of future therapies, which may reduce the burden of current therapies but maintain high efficacy/safety profile, on the human and economic burden of AMD in Spain.

## Supplementary Information

Below is the link to the electronic supplementary material.

Supplementary file1 (DOCX 14 KB)

Supplementary file2 (DOCX 22 KB)

## Data Availability

The datasets used and/or analyzed during the current study are available from the corresponding author on reasonable request.
