# Supplementary material for: Economic burden of age-related macular degeneration in routine clinical practice: the RAMDEBURS study
Source: Int Ophthalmol. 2021 Jun 10;41(10):3427–36. doi: 10.1007/s10792-021-01906-x (PMC8450213; doi:10.1007/s10792-021-01906-x)
Supplement: Supplementary file 1 — Supplementary file1 (DOCX 14 KB) [file 10792_2021_1906_MOESM1_ESM.docx]

**Annex I**. Study centers and investigators of the Real-World Evidence study of patients with Age-Related Macular Degeneration to evaluate the Economic Burden (RAMDEBURS) study group.

**Centers (listed in alphabetical order**):

Bellvitge University Hospital (Barcelona, Spain); Puerta de Hierro-Majadahonda University Hospital (Madrid, Spain); Río Ortega University Hospital (Valladolid, Spain); Santiago de Compostela University Hospital Complex (A Coruña, Spain); University and Polytechnic Hospital of La Fé (Valencia, Spain).

**Investigators (listed in alphabetical order):**

Maximino J Abraldes^1^ (Santiago de Compostela University Hospital Complex. A Coruña, Spain); Luís Arias^1^ (Bellvitge University Hospital. Barcelona, Spain); Marina Aguilar^2^ (University and Polytechnic Hospital of La Fé. Valencia, Spain); Elena Almazán^2^ (Puerta de Hierro-Majadahonda University Hospital, Madrid, Spain); Clara Bastons^2^ (Bellvitge University Hospital. Barcelona, Spain); Cecilia Díez^2^ (Río Ortega University Hospital. Valladolid, Spain); Beatriz Fernández^2^ (Santiago de Compostela University Hospital Complex. A Coruña, Spain); Raquel García^2^ (Río Ortega University Hospital. Valladolid, Spain); María L Gómez^2^ (Santiago de Compostela University Hospital Complex. A Coruña, Spain); Inés Hernández de los Reyes^2^ (Puerta de Hierro-Majadahonda University Hospital, Madrid, Spain); Eugénia Moix^2^ (Bellvitge University Hospital. Barcelona, Spain); Javier Montero^1^ (Río Ortega University Hospital. Valladolid, Spain); José M Ruiz-Moreno^1^ (Puerta de Hierro-Majadahonda University Hospital, Madrid, Spain); Patricia Udaondo^1^ (University and Polytechnic Hospital of La Fé. Valencia, Spain); Carla Villena^2^ (University and Polytechnic Hospital of La Fé. Valencia, Spain).

^1^ Principal Investigator

^2^ Subinvestigator
