# Supplementary material for: Economic burden of age-related macular degeneration in routine clinical practice: the RAMDEBURS study
Source: Int Ophthalmol. 2021 Jun 10;41(10):3427–36. doi: 10.1007/s10792-021-01906-x (PMC8450213; doi:10.1007/s10792-021-01906-x)
Supplement: Supplementary file 2 — Supplementary file2 (DOCX 22 KB) [file 10792_2021_1906_MOESM2_ESM.docx]

**Annex II**. Overview of the unit costs (€). This information has been updated for the year 2020 [20].

| **Antiangiogenic treatments** | **Unit costs (€)**  **(€, 2020)** | **Comments** |
| --- | --- | --- |
| Bevacizumab [21]* | 50.00 | 40 injections/vial |
| Aflibercept [22]* | 247.33 | 3 injections/vial |
| Ranibizumab [23]* | 742.00 | One injection/ vial or pre-filled syringe |
| Sanitary and consumable supplies | 9.43/injection |  |
| **Diagnostic examinations** | **Costs (€)** | **Comments** |
| OCT | 123.01 |  |
| FA | 65.39 |  |
| ICG | 86.47 |  |
| Autofluorescence | 39.78 | Associated with fundus examination |
| Retinography | 140.19 |  |
| Fundus^1^ | 15.92 |  |
| Fundus^2^ | 53.05 |  |
| Visual acuity | 53.05 |  |
| Tonometry | 53.05 |  |
| Genetic test | 60.96 | Associated with GGT |
| Angio-OCT | 136.83 | Estimated according to OCT |
| Blood analysis | 62.84 |  |
| Urine analysis | 54.05 |  |
| Visual field | 46.47 |  |
| Ultrasonography | 65.67 |  |
| **Medical examinations** | **Costs (€)** | **Comments** |
| Ophthalmology | 77.50 |  |
| Emergency | 139.71 |  |
| **Surgery** | **Costs (€)** | **Comments** |
| Vitrectomy | 2,329.07 |  |

* Cost per injection

1. Indirect ophthalmoscopy

2. Biomicroscopy

Abbreviations:

OCT: Optical coherence tomography; FA: Fluorescein angiography; ICG: Indocyanine green; GGT: Glaucoma genetic test.
